# Mechanism of and Threshold Biomechanical Conditions for Falsetto Voice Onset

**DOI:** 10.1371/journal.pone.0017503

**Published:** 2011-03-07

**Authors:** Shinji Deguchi

**Affiliations:** Department of Biomedical Engineering, Tohoku University, Aramaki-Aoba, Sendai, Japan; University of Manchester, United Kingdom

## Abstract

The sound source of a voice is produced by the self-excited oscillation of the vocal folds. In modal voice production, a drastic increase in transglottal pressure after vocal fold closure works as a driving force that develops self-excitation. Another type of vocal fold oscillation with less pronounced glottal closure observed in falsetto voice production has been accounted for by the mucosal wave theory. The classical theory assumes a quasi-steady flow, and the expected driving force onto the vocal folds under wavelike motion is derived from the Bernoulli effect. However, wavelike motion is not always observed during falsetto voice production. More importantly, the application of the quasi-steady assumption to a falsetto voice with a fundamental frequency of several hundred hertz is unsupported by experiments. These considerations suggested that the mechanism of falsetto voice onset may be essentially different from that explained by the mucosal wave theory. In this paper, an alternative mechanism is submitted that explains how self-excitation reminiscent of the falsetto voice could be produced independent of the glottal closure and wavelike motion. This new explanation is derived through analytical procedures by employing only general unsteady equations of motion for flow and solids. The analysis demonstrated that a convective acceleration of a flow induced by rapid wall movement functions as a negative damping force, leading to the self-excitation of the vocal folds. The critical subglottal pressure and volume flow are expressed as functions of vocal fold biomechanical properties, geometry, and voice fundamental frequency. The analytically derived conditions are qualitatively and quantitatively reasonable in view of reported measurement data of the thresholds required for falsetto voice onset. Understanding of the voice onset mechanism and the explicit mathematical descriptions of thresholds would be beneficial for the diagnosis and treatment of voice diseases and the development of artificial vocal folds.

## Introduction

**Figure 1:**
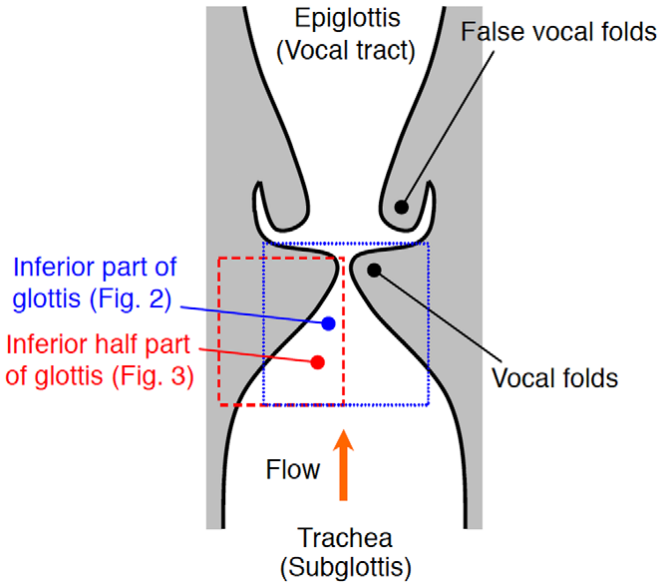
Schema of the front section of the entire glottis.

**Figure 2:**
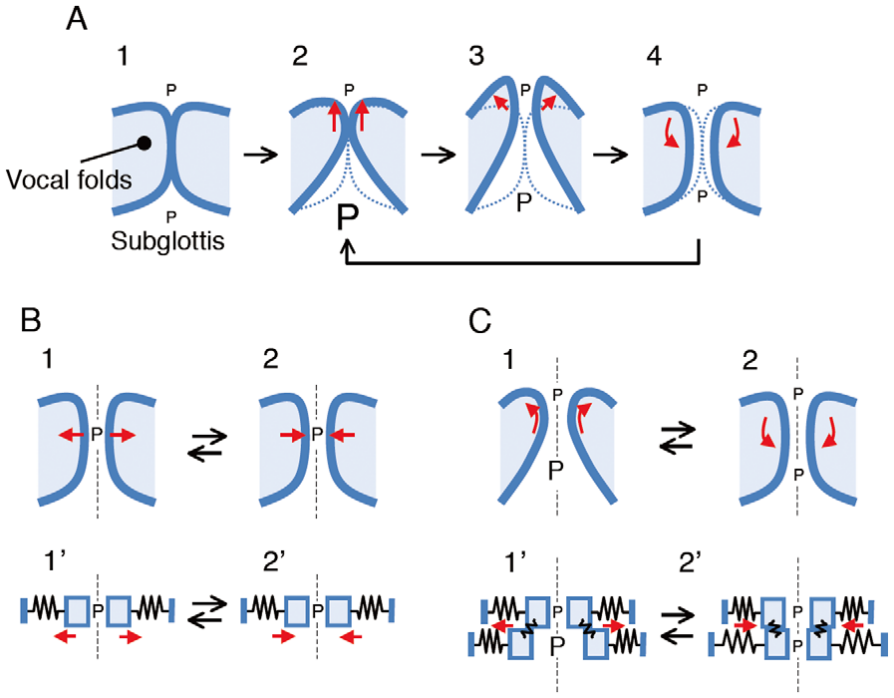
Relationship between the glottal pressure and vocal fold deformation. (A) The positive energy transfer from airflow to vocal fold motion with glottal closure or collision, previously submitted by van den Berg [9] as the myoelastic-aerodynamic theory. The inferior half of the glottis (dashed rectangular region in Fig. 1) is modeled. The theory suggests that the vocal folds are initially sucked together due to the Bernoulli effect. Alternatively, the vocal folds may initially be closed due to laryngeal muscle contraction without the help of the fluid (1). In either case, the glottal closure increases the subglottal pressure, resulting in upward deformation of the vocal folds (2). Here, the size of the letter *P* indicates the magnitude of the glottal pressure, and the dashed lines indicate the original position of the vocal folds. The pressure is more or less diminished due to the restart of the flow after the reopening of the glottis (3), which then allows the vocal folds to return to the original position due to elastic recoil (4). (B and C) Mucosal wave-based explanation for self-excitation of vocal fold motion without closure and the resultant drastic increase in the subglottal pressure. Net positive energy transfer is not achieved when a single degree of freedom model is employed (B) while it may be possible for a model with a degree of freedom of more than two (C). However, the schema (C) assumes a quasi-steady flow assumption, which is not applicable to a high frequency range such as that for a falsetto voice. For details, see the text.

**Figure 3:**
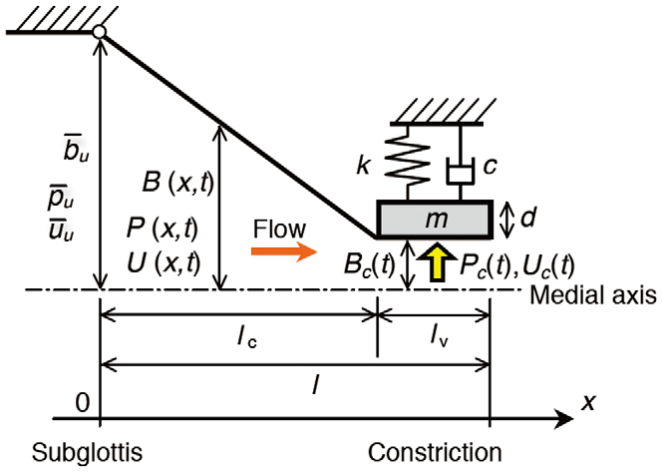
Vocal fold model. The inferior half of the glottis (dashed rectangular region in Fig. 1) is modeled.

**Figure 4:**
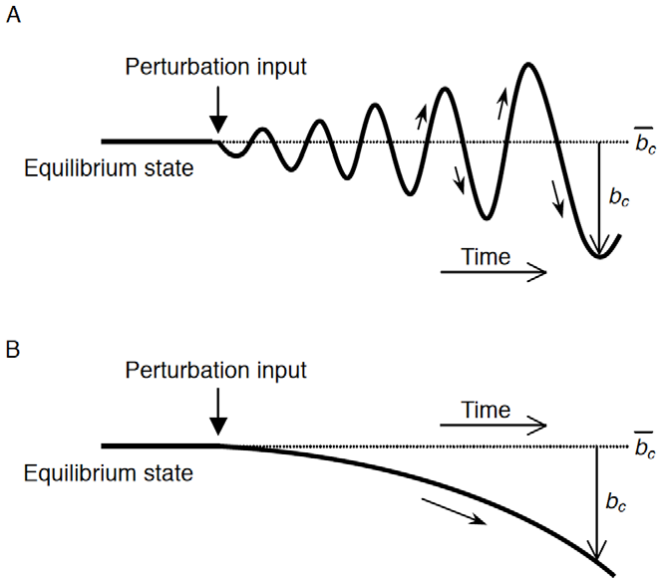
Schema of two types of instability. Flutter or oscillatory divergence (A). This dynamic instability results in the self-excitation of the vocal folds. Divergence or static instability (B).

The self-excited oscillation of the vocal folds located at the larynx produces the major sound source of a voice [1]–[8]. This self-excitation is caused by the flow-structure interaction between respiratory airflow and vocal fold tissue. During self-excitation, airflow must provide the energy necessary for the development of vocal fold oscillation, otherwise, the vocal fold motion decays with time owing to frictional damping in the tissue [4], [5]. Van den Berg [9] provided the first mechanics-based explanation for the voice onset mechanism. He argued in his myoelastic-aerodynamic theory that a pressure drop across the constricted glottis (i.e., the flow path formed by a pair of vocal folds; see Fig. 1), which is created by the Bernoulli effect, sucks the vocal folds together and closes the glottis. Alternatively, the glottis may initially be closed only by laryngeal muscle contraction without the help of flow (see Fig. 2A, 1) [10]. A drastic increase in the subglottal pressure up to the lung pressure, accumulated beneath the closed glottis, pushes the vocal folds downstream and eventually opens the glottal width (Fig. 2A; 2, 3). The blown apart vocal folds are then able to return to their original position owing to an elastic restoring force because the surrounding air pressure at this stage must be relatively low due to the restart of the flow after the glottal opening (Fig. 2A; 3, 4), thereby creating repeated open-close movements. Thus, the vocal fold closure ensures a requirement for self-excitation, i.e., a continuous energy transfer from the flow to the vocal folds.

Then, the question arises as to how glottal pressures asymmetric in magnitude over one oscillatory cycle (i.e., larger in the opening phase than in the closing phase, resulting in a net positive energy transfer) are created with no such glottal closure during vocal fold oscillations [5]. This requirement may not be satisfied in the absence of glottal closure because the Bernoulli effect, whose magnitude is determined by the absolute value of the width or cross-sectional area (but not its time rate of change), has the same value whether the glottis is opening or closing; therefore, the same pressure in magnitude may be applied to the vocal folds, resulting in the failure of developing oscillations (see Fig. 2B) [5]. Note that the vocal folds exhibit wavelike motion in the coronal plane during speech [9], [11]. On the basis of an analytical two-mass vocal fold model, Ishizaka and Matsudaira [1], [2] demonstrated that such wavelike motion or phase lag in motion between the upper and lower masses of the vocal folds led to self-excitation through flow-structure interaction (see Fig. 2C). Titze [4], [5] more explicitly highlighted the role of a mucosal wave while explaining that the vocal folds could experience higher pressure during the opening phase owing to the wavelike motion with the glottis opening at the bottom (upstream) first and then at the top (downstream) and likewise closing at the bottom first and then at the top (see Fig. 2C).

Then, how are these theories related to the actual phonation involving two main vocal registers, i.e., modal and falsetto? In the modal register, oscillating vocal folds (observed with a laryngo-stroboscopy or high-speed camera) entirely close the glottis in each oscillation cycle while deforming with the entire body [9]. Therefore, the mechanism of the modal or normal voice with such pronounced glottal closure is basically explained by the myoelastic-aerodynamic theory (Fig. 2A), which is analogous to the airflow-induced buzzing of the lips that also results in an intermittent outflow [12]. In contrast, the vocal folds in the falsetto register often keep the glottis open while oscillating, through this opening a certain volume of airflow continuously escapes. The myoelastic-aerodynamic theory is not applicable to such oscillations without complete glottal closure (Fig. 2B) [5]. The pioneering analytical models, provided by Ishizaka and Matsudaira [1], [2] and by Titze [5] in the mucosal wave theory (Fig. 2C), were intended to elucidate the onset mechanism for such small amplitude oscillations without complete glottal closure. Then, does the mucosal wave theory describe the falsetto voice mechanism precisely? It is noteworthy that during falsetto phonation, the vocal folds do not always exhibit mucosal wave motions [9], [13]–[19]. The main body of each fold, which consists of the thyroarytenoid muscle, is more or less relaxed in falsetto [5], [11]. The pull of the tissue by the cricothyroid muscle activation thins the vocal folds [9]. Therefore, only the ligamentous superficial layers of the vocal folds enter into vibration, possibly resulting in a negligible mucosal wave. Critical involvements of flow separation mobility in the oscillation mechanisms have been proposed [20]–[25]; however, it is not clear if the reported flow separation behavior is likely in the falsetto voice production with such a negligible vertical motion of the thin vocal folds.

More importantly, although the mucosal wave theory has been the cornerstone of nearly all subsequent theoretical developments of voice mechanics [e.g.], [ 26]–[30], it was developed under a quasi-steady flow assumption [8]. In particular, steady mass conservation and Bernoulli equations [1]–[5] were applied in those analytical studies without experimental verification of the decisive assumption under oscillating conditions. However, recent experimental and theoretical studies have demonstrated that the pressure distribution along the glottis essentially differs from that in a static condition in a range of high but realistic voice fundamental frequencies [23], [31]–[33]. In particular, our group analytically explained that the flow behavior in oscillating constriction (such as the pressure amplitude and phase difference between related variables) depends on the Strouhal number, i.e., a dimensionless number describing which effect is stronger, a flow induced by rapid wall motion (numerator) or a flow induced by the convective acceleration such as the speeding up of the airflow entering the converging glottis (denominator) [33]. The Strouhal number should not be negligible in the high vocal frequency range. In fact, Ishizaka and Matsudaira [1] described that their theory aimed at revealing self-excited oscillations in the chest register (i.e., a modal voice), possibly implying that the Bernoulli effect-based explanation might not be effective in the falsetto register.

It is also known that air column oscillation in the axially long or narrow vocal tract (including the pharynx and oral cavity) could play an additional role in producing higher pressure in the opening phase than in the closing phase [1]–[5], [34]. However, the threshold lung pressure due to the acoustic coupling between the vocal tract and glottis derived by Titze [4, Equation (50)] was not a function of the glottal width, suggesting that the acoustic coupling may only assist other primary mechanisms in reaching the oscillation threshold. Indeed, falsetto-like oscillations are produced even without a vocal tract in both self-excited physical vocal fold models and excised larynx models [35]–[39]. These observations suggest that the mucosal wave and vocal tract response may not perform a critical role in the onset mechanism of falsetto voice production [1].

In our previous theoretical work on fluid-structure interaction in the glottis oscillating at high speeds, we analytically derived the relationship between the time-varying glottal width and pressure perturbation from general unsteady flow equations [33]. The study demonstrated that a convective acceleration (i.e., a change in velocity over position) of a flow that was originally induced by rapid wall movement becomes comparable in magnitude to the Bernoulli effect within a physiological frequency range typical for a falsetto voice (e.g., >400 Hz). Because of this unsteady flow effect associated with the considerable Strouhal number, which was not taken into account in previous analytical studies on the phonation onset [e.g.], [ 1], [4,27], a phase difference (or time lag) can appear between the vocal fold motion and glottal pressure fluctuation. The time lag in the driving force may thus meet the requirements for self-excitation, i.e., continuous energy transfer from the airflow to vocal fold motion. Thus, we suggested that self-excitation could possibly occur from the inherent glottal flow property independent of the mucosal wave motion or inertial acoustic loading from the vocal tract or subglottis [40].

In our unsteady flow theory, we linearized the flow descriptions through perturbation analysis around the time-mean value; these small amplitude assumptions were also used in the previous analytical studies by Ishizaka and Matsudaira [1], [2] and Titze [4], [5]. While sustained modal voice in general involves large amplitude motion of the vocal folds with collision, the linearized small amplitude restriction indicates that the results are applied only to analyses of the phonation onset with no glottal closure. A falsetto voice often has a simple sinusoidal sound waveform with few higher harmonic waves, even in sustained conditions [9], indicating that the vocal fold oscillation in a falsetto voice occurs only near the surface tissue with small amplitudes. In addition, the fundamental frequency of a falsetto voice is sufficiently high to provoke the phase alteration caused by the unsteady flow effect [33]. Our flow theory thus seems to fit well with the analysis of the falsetto voice onset.

In the present study, we employed this flow theory to analytically derive the threshold conditions required for self-excitation of the vocal folds. The analysis demonstrated that an unsteady flow effect, or more specifically a convective acceleration of a flow induced by rapid wall movement, provides negative damping at the critical subglottal pressure or volume flow, inducing self-excited oscillation reminiscent of falsetto voice onset. Large amplitude behavior associated with established limit cycles, which involves nonlinear effects such as register transition and onset/offset hysteresis [27], [30], [41], [42], will not be discussed in this study. The tools of analysis for such large amplitude oscillations are primarily numerical [6], [7], [43], [44]. It is sometimes elusive to intuitively understand our ability to speak with a falsetto voice from such numerical (computational) results and the criteria for estimating the efficiency of voice production. Thus, it would be appropriate to devote a separate study to such numerical analyses.

## Analysis

### Model geometry

The position along the inferior half of the glottis (see Fig. 1) is given by spatial coordinate *x* (see Fig. 3). Each cross section is assumed to be rectangular with a constant vocal fold length *l*_g_ (normal to the plane of the paper). The vocal fold shape and its motion are assumed to be symmetrical with respect to the medial axis. The glottal half-width *B*(*x*,*t*) perpendicular to the *x* direction where *t* represents the time is assumed to be a constant value 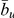 at the upstream inlet (*x* = 0) and a time-varying value *B*(*l_c_*,*t*) (rewritten as *B_c_*(*t*)) at the entrance of the narrowest constriction (*x* = *l*_c_). The glottis between *x* = 0 and *l*_c_ is simply connected by a straight line:
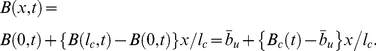
(1)The vocal fold constriction (from *x* = *l_c_* to *l_c_*+*l_v_*) is assumed to be a parallel path where *l_v_* is the vocal fold thickness. In fact, the linear geometry of the glottis presented here is not a requirement of the current study, rather, it is assumed to simplify analytical integration calculations and does not essentially affect the onset mechanism described below.

### Flow model

In our previous work, we developed a one dimensional unsteady flow theory that explicitly described the relationship between the time-varying glottal width and fluid pressure [33]. As this theory was applied to the present analysis, its derivation is briefly shown here with modifications to correspond with the present flow channel geometry. According to the accumulated knowledge on the internal flow in collapsible tubes [46]–[53], the mass and momentum conservation equations for an unsteady, viscous, and incompressible flow in a deformable tube are described by
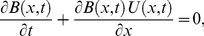
(2)

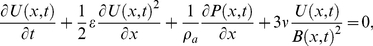
(3)respectively, where *U*(*x*,*t*) is the velocity, *P*(*x*,*t*) is the pressure, *ε* is a factor related to flow separation and the vena contracta [3], [48], [53], *ρ*_a_ is the air density, and *ν* is the kinematic viscosity of air. Note that hereafter, for free variables *x* and *t*, the parentheses are often omitted for notational simplicity. Each variable is decomposed into a time-averaged component (lowercase letter with an overbar) and a time-varying perturbation component (lowercase letter):
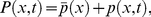
(4)

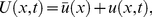
(5)

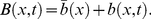
(6)The perturbations are small, and the terms of order higher than the quadratic are neglected. We assumed that pressure and velocity at *x* = 0 have constant values 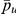 and 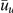, respectively (see Fig. 3). The fixed boundary condition at the inlet is consistent with the previous analytical studies that assumed an ideal constant pressure source [4]. It is assumed that the fluid pressure at the center of the narrowest constriction, *x* = *l*_c_+*l_v_*/2, drives the vocal fold motion, as considered later in a vocal fold model with a single degree of freedom that does not exhibit mucosal waves. At the driving point,
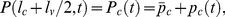
(7)

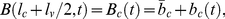
(8)where *B_c_*(*t*) = *B*(*l_c_*,*t*) = *B(l_c_*+*l_v_*/2,*t*) (see Fig. 3). Equation (2) is integrated along *x* from 0 to *l*_c_+*l_v_*/2, yielding a perturbation fluid velocity at the driving point [33]:
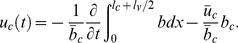
(9)The perturbation glottal pressure that interacts with the perturbation of the vocal fold displacement is:
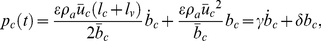
(10)where a dot over a variable denotes its time derivative: 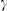 and *δ* are defined as the coefficients of each term. The first term 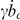 represents a force due to the convective acceleration (i.e., the effect of time-independent acceleration of a fluid with respect to space) of the wall motion-induced flow [33]. The second term *δb_c_* represents a convective inertial force, i.e., the Bernoulli effect. The former unsteady term could become comparable in magnitude to the latter steady term when the wall moves quick enough to produce a considerable wall motion-induced flow that is distinct from the steady flow determined by the static geometry of the channel. The considerable unsteady effect causes a phase lag between the pressure *p_c_* and the motion *b_c_*, which could result in meeting the requirements for self-excitation described above. The coupling of Equations (2) and (3) should actually have seven perturbation terms in general, but Equation (10) has only two terms because the remaining five terms, including the effect of air viscosity on the perturbation glottal pressure, are negligibly small in magnitude as confirmed by a thorough scale analysis performed in our previous work [33].

Volume flow is defined by:
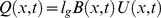
(11)and divided depending on whether it is time dependent:
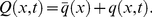
(12)Temporal averages of Equations (2) and (3) satisfy:
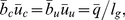
(13)

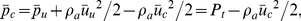
(14)where *P_t_* is the subglottal pressure (shown by the total pressure). Equation (14), obtained with simplification based on scale analysis [33], corresponds to Bernoulli's law for steady flow.

### Vocal fold model

The equation of motion for the vocal fold is given by that of a mass-spring-damper oscillator in a lumped element representation (see Fig. 3):
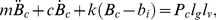
(15)where 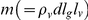, 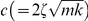, *k*, *b_i_*, 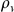, *d*, and 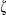 are the mass, damping coefficient, spring constant, initial half-width, density, depth, and damping ratio of the vocal fold, respectively. The depth *d* is not attributed to any one histological component such as the ligament, but represents the composite depth of effective structures; it is introduced to determine the effective mass *m* involved in vibration, which is a function of the density 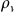 as well as the geometric parameters, i.e., the vocal fold depth *d*, thickness *l_v_*, and length *l_g_*. In the steady state, Equation (15) becomes:
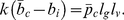
(16)From Equations (7), (8), (10), and (15), we obtain the following perturbation equation:
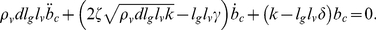
(17)The coefficients of the second and third terms on the left side represent effective damping and effective stiffness, respectively.

### Threshold pressure and volume flow for oscillatory divergence due to negative damping

The system of Equations (13), (14), and (16) that describes steady states has, in general, three analytical solutions (one trivial solution for the hydrostatic condition and two non-trivial solutions with non-zero flow velocities). However, in the actual vocal fold mechanics because flow accelerates significantly in the constricted glottis, the following approximations are applicable [4]:
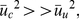
(18)

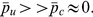
(19)Here, 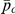 is assumed to be zero to exclude acoustic coupling with the vocal tract. With these approximations, the steady state has the following single solution with real numbers:
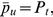
(20)

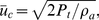
(21)

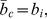
(22)

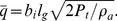
(23)When the coefficient of the second term of Equation (17) is negative (i.e., negative damping), a dynamic instability or flutter [49], [54] occurs in which a perturbation gradually develops to induce oscillatory divergence (see Fig. 4A). The threshold subglottal pressure 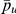 and volume flow 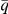 for flutter are explicitly described by:
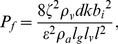
(24)

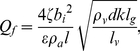
(25)respectively, where the constriction length *l* is defined as
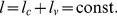
(26)The critical subglottal pressure described by Equation (24) is interpreted as the minimum lung pressure required for vocal fold oscillation onset. Such a critical lung pressure is in general called the phonation threshold pressure (PTP) and is potentially useful in diagnosis to noninvasively evaluate vocal fold stiffness and quantify the ease of phonation [4], [55]. Equation (25) denotes the minimum volume flow necessary for voice onset, which has recently been called the phonation threshold volume flow (PTF) [26], [56], [57]. As mentioned in Section 2.2, we estimated in a previous work that the viscous resistance in the glottis has a negligibly small magnitude compared to the convective and unsteady flow effects [33]. This scale analysis yields Equation (14) or the Bernoulli's equation, implying that the fluid energy (i.e., the sum of the pressure and kinetic energies) is conserved. PTP (*P_f_*) and PTF (*Q_f_*) are obtained from the same condition (i.e., the second term of Equation (17) is zero), that is, they are merely one expression of the same critical condition required for the oscillatory divergence. Hence, in the present modeling, the input of fluid energy to the vocal fold system to achieve PTP is the same as that for PTF.

### Unidirectional divergence due to negative stiffness

When the coefficient of the third term of Equation (17), representing the intrinsic stiffness versus the Bernoulli effect, is negative (i.e., negative stiffness), a static instability or divergence [54] occurs upon imposition of a perturbation to the system (see Fig. 4B) [58]. The threshold subglottal pressure and volume flow for the divergence are described by:
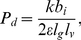
(27)

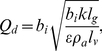
(28)respectively. The present study employed a simple single degree of freedom model for the vocal folds (see Fig. 3) to exclude the mucosal wave motion. In addition, we linearized the behavior around the time-mean value. Within this current modeling, the static instability indicated that a unidirectional deformation occurred around the slightly abducted (spread apart) vocal folds, and the vocal folds were either blown open or closed by the airflow [58]. Note that if a vocal fold model with a degree of freedom more than two is employed together with proper mechanical properties [3], limit cycles (different from being kept opened or closed) after the static instability could be established in a collision-dependent manner explained by the myoelastic-aerodynamic theory (see Fig. 2A). To determine whether the limit cycles occur after the static instability (as already performed by Ishizaka and Flanagan [3]), incorporation of the following is required: (1) an additional degree of freedom to the vocal fold model, (2) another mechanical property related to the coupling between the upper and lower masses (or the ease of mucosal wave propagation), and (3) the effect of glottal closure or collision. Such additional modeling is beyond the scope of the present study that aims at introducing a framework of the basic principles to help in understanding the mechanics of falsetto voice onset. In addition, the limit cycles with collision after static instability are in general regarded as the source of modal voice [9], [58]. Therefore, henceforth we focus on only negative damping-induced flutter, i.e., *P_f_* (PTP) and *Q_f_* (PTF), but not on static instability *P_d_* and *Q_d_*. In fact, Ishizaka and Matsudaira [1] and Titze [4] also developed their theories on the basis of negative damping-induced oscillatory divergence.

### The effect of vocal fundamental frequency on PTP and PTF

PTP and PTF expressed by Equations (24) and (25), respectively, contain the spring constant *k*, representing the intrinsic stiffness of the vocal folds, which may be difficult to measure at various conditions [4], [28]. In terms of practical use, therefore, the fundamental frequency of a voice *F*_0_ may be suitable as an alternative dependent variable instead of *k*. Thus, another form of PTP and PTF as a function of *F*_0_ is derived below. At the critical condition that allows the onset of flutter, the coefficient of the second term of Equation (17) becomes zero. The frequency at that instant is defined as:
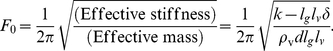
(29)From Equations (10) and (21), the Bernoulli effect 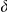 at the oscillatory divergence onset (i.e., *P_t_* = *P_f_*) is:
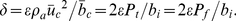
(30)From Equations (29) and (30),
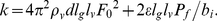
(31)Substitution of Equation (31) into Equations (24) and (25) yields alternative forms of PTP and PTF that contain *F*_0_ as an explicit factor:
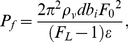
(32)

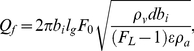
(33)where
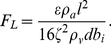
(34)From Equations (10), (17), (21), (22), and (26), the newly introduced dimensionless number *F_L_*, associated with the ease of flutter [49], [54] in a viscoelastic flow channel or falsetto voice onset, is interpreted as:
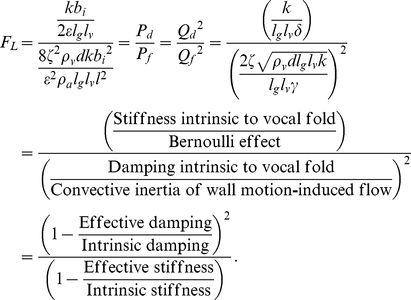
(35)Thus, *F_L_* quantifies the relative importance of the distinct effects. Note that as long as flutter occurs more readily than the unidirectional divergence given by Equations (27) and (28), *P_f_*<*P_d_*, and hence, *F_L_*>1. Thus, both *P_f_* and *Q_f_* have positive values by definition.

### Quantitative assessment

**Figure 5:**
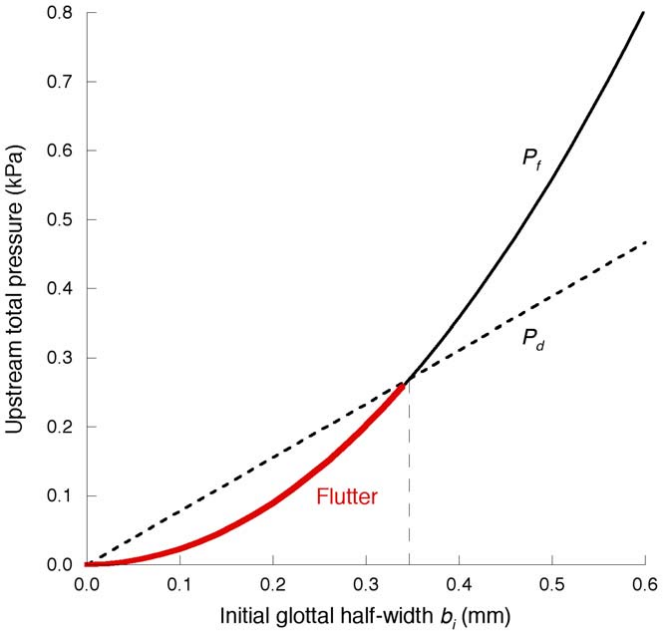
*P_f_* (PTP) versus initial glottal half-width *b_i_*. For comparison, *P_d_* is also shown. When the upstream total pressure reaches a value within the red curve, flutter or self-excited oscillation of the vocal folds occurs. The parameter values employed are described in Section 2.5.

**Figure 6:**
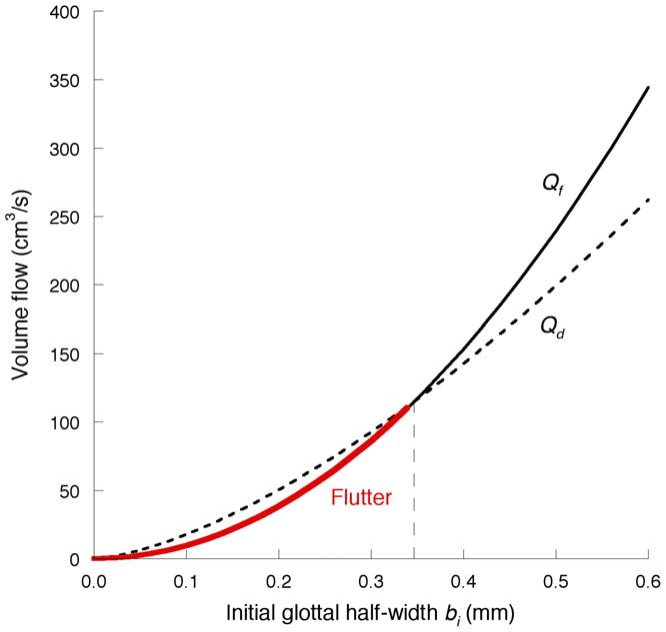
*Q_f_*(PTF) versus initial glottal half-width *b_i_* shown by the red curve.

To evaluate the quantitative validity of the derived PTP (*P_f_*, Equation (24)) and PTF (*Q_f_*, Equation (25)), we applied the following representative values, which are within the physiological range used in the previous phonation modeling [2], [3], [28], [59]–[61]: 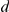 = 0.35 mm, 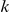 = 35 N/m, 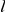 = 10 mm, 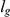 = 15 mm, 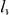 = 1.5 mm, 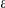 = 1, 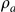 = 1.1 kg/m^3^, 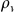 = 1.02×10^3^ kg/m^3^, and 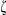 = 0.235. PTP and PTF, shown as functions of the initial glottal half-width *b_i_*, were estimated as ∼0.1–1 kPa and ∼10–300 cm^3^/s, respectively (see Figs. 5 and 6). They were comparable in magnitude as per the reported measurement data [26], [42], [55]–[57], [62]–[64]. Note that on the basis of his measurements, van den Berg [9] mentioned that 9 to 10 mL of air were sufficient for the production of sound for 0.5 s (i.e., roughly equal to 20 cm^3^/s).

For comparison, the critical values for the static instability (*P_d_*, Equation (27); *Q_d_*, Equation (28)), which might be related to modal voice production [2] as discussed in Section 2.5, are also shown in Figures 5 and 6 as functions of *b_i_*. At a small *b_i_* of <0.35 mm, flutter requires a lower upstream pressure or volume flow as compared with that of the static instability, indicating that the former occurs more readily. On the other hand, the static instability occurs more readily than the flutter at a high *b_i_*.

**Figure 7:**
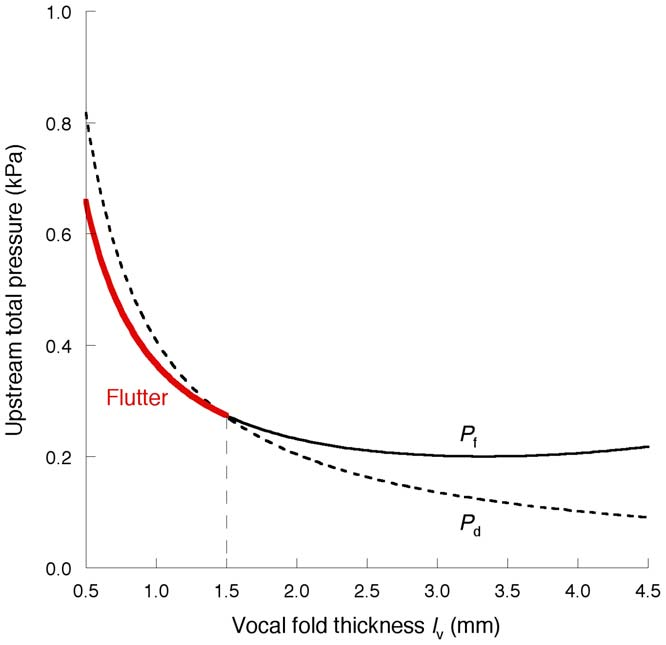
*P_f_* (PTP) versus initial vocal fold thickness *l_v_* shown by the red curve.

**Figure 8:**
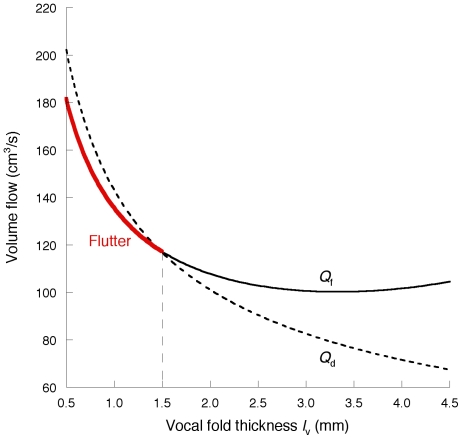
*Q_f_* (PTF) versus initial vocal fold thickness *l_v_* shown by the red curve.

Figures 7 and 8 depict the effect of vocal fold thickness *l_v_* on PTP and PTF, respectively, at a constant *b_i_* of 0.35 mm. As the thickness is reduced, it becomes more difficult to initiate flutter, but it is more likely to occur than static instability at thin vocal folds. This tendency is consistent with the morphological observation that in the falsetto voice, the vocal fold tissue margins are rather thin and pointed due to the tension exerted by the contraction of the cricothyroid muscle [9], [59].

**Figure 9:**
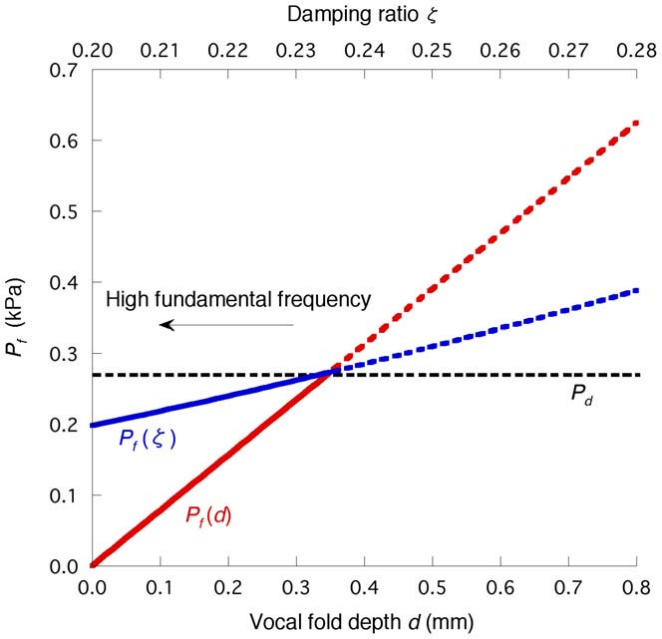
The effects of vocal fold depth *d* and damping ratio 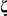 on *P_f_*. To occur, flutter requires a lower input pressure than the static instability (*P_d_*) at a low *d* or 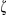 range, suggesting that flutter will appear at a high fundamental frequency range.

*P_f_* and *P_d_* are also shown as functions of either vocal fold depth *d* or the damping ratio 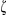 with a constant *b_i_* of 0.35 mm (see Fig. 9). As described in the introduction and in the literature [9], [60], activation of the cricothyroid muscle at a high fundamental frequency reduces the vocal fold depth. Thus, the depth *d* can correspond to the effective tissue depth of vibration [60]. In general, muscular tissues have a larger hysteresis loss between loading and unloading than ligamentous tissues [65], [66]. Therefore, the decrease in the depth that may alter the dominant vibrating part from the muscular region to the ligamentous one [4], [9] would be followed by a reduction in the damping ratio of related tissues. Since the two parameters *d* and 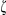 do not affect the magnitude of *P_d_* (Equation (27)), *P_f_* falls below *P_d_* as the expected cricothyroid muscle activation proceeds. Thus, the quantitative evaluation suggests that flutter occurs more readily than static instability at a high fundamental frequency as in the falsetto voice (see Fig. 9).

**Figure 10:**
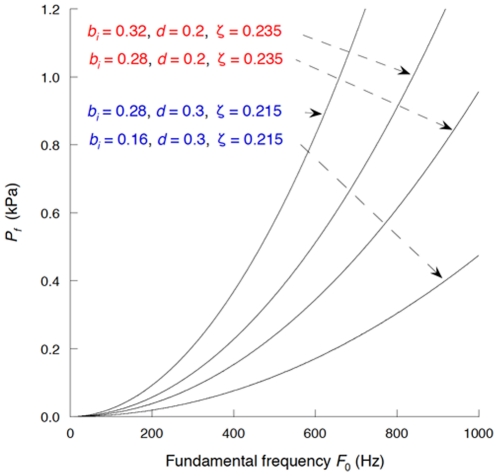
The effect of vocal fundamental frequency *F*_0_ on *P_f_*.

**Figure 11:**
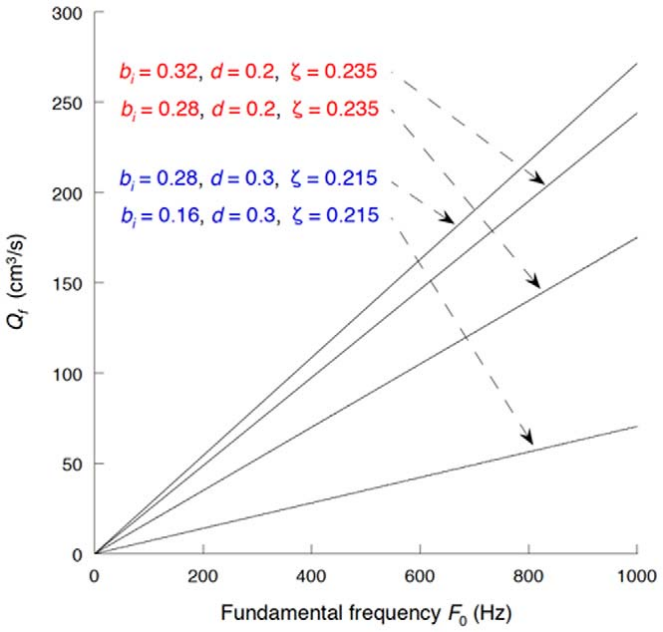
The effect of vocal fundamental frequency *F*_0_ on *Q_f_*.

Figures 10 and 11 or supplementary Figures S1 and S2 display the effects of vocal fundamental frequency *F*_0_ on PTP and PTF, respectively, as functions of the initial glottal half-width *b_i_*, vocal fold depth *d*, and the damping ratio 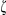. In these figures, the initial glottal half-width *b_i_* is fixed at less than 0.35 mm, assuring that flutter occurs (see Figs. 5 and 6). *P_f_* is proportional to the square of *F*_0_ as explicitly shown in Equation (32), whereas *Q_f_* is proportional to the first power of *F*_0_ as shown in Equation (33). This quantitative evaluation demonstrates that PTP and PTF have realistic values within the frequency range typical for a falsetto voice (i.e., >400 Hz) [26], [55].

## Discussion

Thus far, numerical (computational) simulations of falsetto-like voice production have been performed using unsteady flow equations, essentially the same as those used in the present study [6], [7], [43]–[45]. Such numerical analyses are useful in investigating the entire process of the voice production over a wide range where nonlinear effects may play essential roles. In contrast, the present study provided the first complete analytical description dealing with the mechanism of self-excitation at high fundamental frequencies, reminiscent of falsetto voice onset. The explicit descriptions of the phonation onset (*P_f_* and *Q_f_*; Equations (24) and (25) or (32) and (33), respectively) may be useful for capturing the mutual relationships among basic parameters at a glance and for understanding the essential mechanism of the complex fluid-structure interaction phenomenon. The threshold conditions were quantitatively reasonable in view of the reported measurement data [9], [26], [42], [55]–[57], [62]–[64] (see Figs. 5, 6, 7, 8, 9, 10, 11). The mutual relationships among parameters, such as the effect of vocal fold stiffness on the onset upstream pressure, make intuitive sense and are consistent with the tendencies obtained in previous numerical analyses [6], [7], [43]. The present results were obtained from a single degree of freedom system with a small amplitude approximation. Therefore, the findings derived analytically from general physical governing equations are specifically applicable to a small amplitude or a falsetto voice rather than a modal voice that has large amplitude and non-uniform motion of the vocal folds.

**Figure 12:**
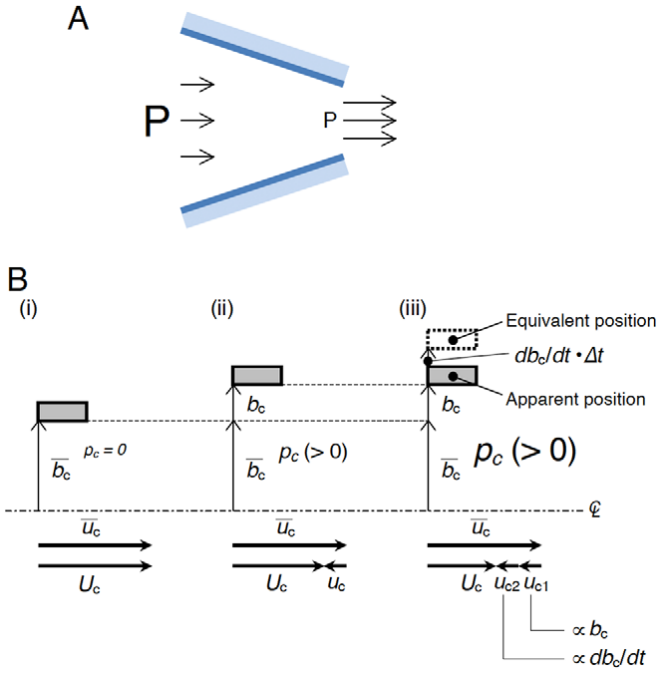
The effect of rapid wall movement on internal flow. Convective acceleration (or the Bernoulli effect) is the time-independent acceleration of a fluid with respect to space (A). The size of the letter *P* and the length of the arrows indicate the magnitude of glottal pressure and that of the fluid velocity in a converging duct, respectively. Although the flow may be steady (time-independent), the fluid accelerates as it moves down the converging duct; thus, there is an acceleration happening over position, referred to as convective acceleration or the Bernoulli effect. Relationship between the glottal width and velocity (B). At a steady state (i), the perturbations of variables *b_c_*, *u_c_*, and *p_c_* are all zero. If a perturbation *b_c_* with a positive value is slowly given to the steady state (ii), such enlargement of the constriction weakens the convective acceleration and hence decreases the instantaneous velocity *U_c_* with the appearance of a negative perturbation velocity *u_c_* and a consequent positive perturbation pressure *p_c_*. Instead, let us consider a case in which a perturbation *b_c_* with a positive value is very quickly given to the steady state (iii). The wall moves so fast that not only the *b_c_*-induced negative perturbation velocity *u_c1_* (identical to *u_c_* in (ii)) but also an additional negative velocity component *u_c2_* proportional in magnitude to the time derivative of the wall motion *db_c_*/*dt* appears. Here, *u_c1_* and *u_c2_* correspond to the second and first terms on the right side of Equation (9), respectively. The additional velocity component due to the rapid wall movement also experiences convective acceleration; therefore, a perturbation pressure higher in magnitude than (ii) is obtained at (iii), as graphically shown by *p_c_* with a big size. Likewise, a fast narrowing of the wall (i.e., negative *b_c_*) yields a negative perturbation pressure *p_c_* whose absolute value is greater than that estimated from the apparent width (figure not shown). Previous quantitative evaluation [33] suggested that the magnitude of *u_c2_*-originated perturbation pressure (i.e., the first term on the right side of Equation (10)) reaches approximately 50% of the *u_c1_*-originated one (the second term on the right side of Equation (10)) at 500 Hz; thus, playing a significant role for driving pressure in falsetto voice onset.

**Figure 13:**
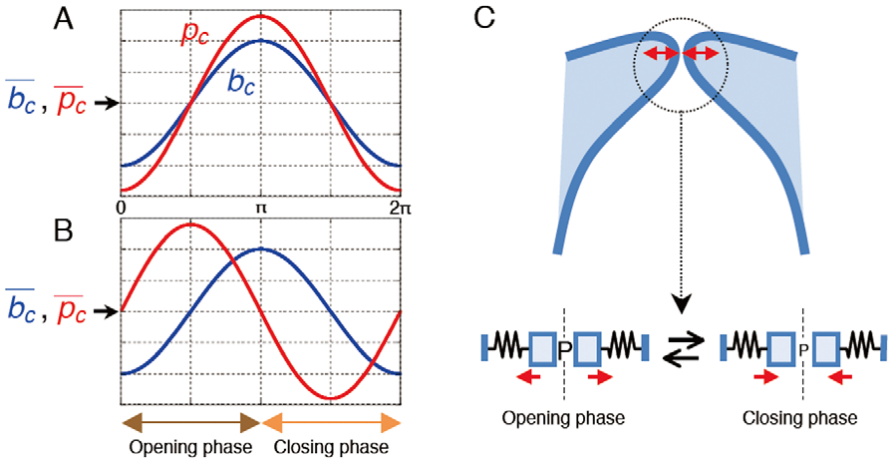
Mechanism of falsetto voice onset. Pressure and width fluctuate with the same phase at a quasi-steady state (A). In contrast, due to the convective acceleration of the rapid wall motion-induced flow shown in (iii) of Figure 12B, a phase difference appears between the variables (B) because the magnitude of *p_c_* is partly determined by how fast the wall moves (Equation (10)). Previous work [33] showed that the phase difference is practically dependent only on the Strouhal number, but note that the perturbation pressures always have (on average) larger values during the opening phase than during the closing phase. This asymmetry in pressure magnitude over one cycle (C) can lead to self-excitation of the vocal folds only if the threshold condition *P_f_* or *Q_f_* is satisfied.

The current study mathematically revealed that the vocal folds could be self-excited independent of a mucosal wave or glottal closure if general unsteady flow equations are employed. Oscillatory divergence or flutter (see Fig. 4A) is thus triggered through a different mechanism than that previously considered. In particular, when the vocal fold wall moves so quickly that the effect of the time-varying motion achieves a significant magnitude, a slower fluid velocity than that estimated based on a steady flow appears in the glottis during the opening vocal fold movement (see Figs. 12A and 12B). This deceleration occurs because individual air particles (but not liquid particles as in Washio *et al.* [67]) adjacent to the wall must follow the quick movement so as to not break the interface between the solid wall and the fluid. This model is essentially different from that of the Bernoulli effect in which the absolute value of the glottal width determines the fluid velocity (see Fig. 12B). Likewise, in the closing phase, a faster fluid velocity than that estimated based on a steady flow is present. In consequence, a higher driving pressure is applied to the vocal folds during the opening movement (see Fig. 13), inducing the self-excited oscillation, because the vocal folds can receive positive energy from the flow as the oscillation proceeds.

The fluid force that induces the static instability (shown by the second term in the middle of Equation (10)) comes from the Bernoulli effect. This term is proportional to the dynamic pressure or the square of the glottal velocity 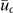. In contrast, the fluid force that induces flutter is caused by the convective acceleration of a fluid velocity induced by rapid motion of the vocal fold tissue (see Fig. 12). The tissue motion-induced velocity itself is independent of the time-mean fluid velocity 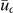 as explicitly described in the first term on the right side of Equation (9). Therefore, the flutter-related force (shown by the first term in the middle of Equation (10)) varies with the first (but not the second) power of 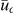, representing the effect of convection. Both the separate fluid forces are inversely proportional to 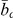 (or *b_i_* of Equation (22)) as shown in Equation (10), because the ratio of the displacement perturbation *b_c_* to the initial width *b_i_* (rather than *b_c_* itself) influences the pressure perturbation *p_c_*. Equations (20) and (21) imply that the critical subglottal pressures that induce instability should be proportional to the square of the glottal velocity 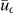. As a result, PTP (i.e., *P_f_*) is proportional to the square of *b_i_* whereas the critical pressure for the static instability (*P_d_*) is proportional to the first power of *b_i_*. Thus, the diverse dependencies on *b_i_* yield flutter- and unidirectional divergence-dominant regions (see Fig. 5). Equations (20) and (23) imply that PTF is proportional to *b_i_* as well as to the square root of PTP, indicating that PTF (*Q_f_*) is proportional to the square of *b_i_*, whereas the critical volume flow for unidirectional divergence (*Q_d_*) is proportional to the two-thirds power of *b_i_* (see Fig. 6).

Although the relationships between *F*_0_ and PTP or PTF are explicitly expressed in Equations (32) and (33) and graphically shown in Figures 10 and 11, we should note that the vocal fold-related parameters that constitute *P_f_* and *Q_f_* are in fact interdependent on each other. Laryngeal muscle activation results in diverse changes in vocal fold geometry and mechanical properties; thus, a shift in one parameter alters multiple interdependent parameters simultaneously. The parameters used in the present modeling are independent of each other in order to avoid expedient assumptions and consequent loss of generality. Establishing a plausible assumption that adequately specifies the relationship between the parameters will be needed for practical cases and will be a subject of future investigations. Note that the current explanation for the falsetto voice onset mechanism does not exclude the effect of acoustic coupling with the axially long vocal tract and subglottis [3]–[5], [34], [40]. The acoustic effect as well as the unsteady flow behavior highlighted here may cooperatively contribute to the self-excitation in actual phonation. However, it is notable that human PTP–*F*_0_ relationships reported by Solomon *et al.* [63] and Titze [62], [68], which were examined in high frequency ranges, indicate that some subjects appear to have a concave PTP–*F*_0_ curve at a high frequency range, thereby being consistent with the current result (see Fig. 10).

In summary, we analytically derived biomechanical conditions required for falsetto voice onset from general unsteady flow equations. In this model, the self-excitation of the vocal folds in a falsetto voice arises through inherent flow properties in a rapidly oscillating wall, a process that is distinct from the mucosal wave-based explanation submitted previously and based on a quasi-steady flow assumption. This model of the falsetto voice onset provides explicit relationships among the vocal fold geometry, biomechanical parameters, and fundamental frequency.

## Supporting Information

Figure S1
**The effect of vocal fundamental frequency** ***F*** **_0_ on** ***P_f_*** **.** Effects of vocal fold depth *d* and damping ratio 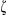 with a fixed initial glottal half-width *b_i_* = 0.32.(TIF)

Figure S2
**The effect of vocal fundamental frequency** ***F*** **_0_ on** ***Q_f_*** **.** Effects of vocal fold depth *d* and damping ratio 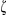 with a fixed initial glottal half-width *b_i_* = 0.32.(TIF)
