# Supplementary figures and images for: Mechanism of and Threshold Biomechanical Conditions for Falsetto Voice Onset
Source: PLoS One. 2011 Mar 7;6(3):e17503. doi: 10.1371/journal.pone.0017503 (PMC3049783; doi:10.1371/journal.pone.0017503)

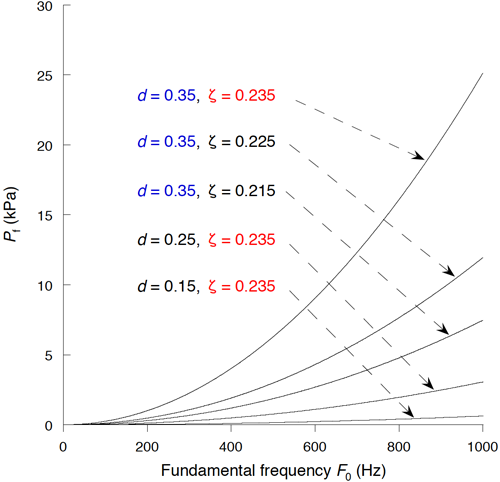

Supplement: Figure S1 — The effect of vocal fundamental frequency F 0 on Pf . Effects of vocal fold depth d and damping ratio with a fixed initial glottal half-width bi = 0.32. (TIF) [file pone.0017503.s001.tif]

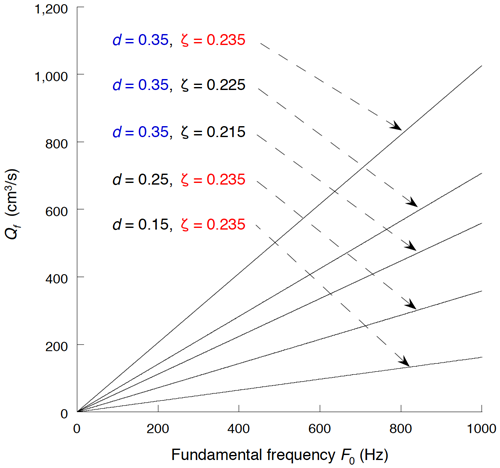

Supplement: Figure S2 — The effect of vocal fundamental frequency F 0 on Qf . Effects of vocal fold depth d and damping ratio with a fixed initial glottal half-width bi = 0.32. (TIF) [file pone.0017503.s002.tif]
